# Nanoconjugates to enhance PDT-mediated cancer immunotherapy by targeting the indoleamine-2,3-dioxygenase pathway

**DOI:** 10.1186/s12951-021-00919-z

**Published:** 2021-06-14

**Authors:** Xueyuan Yang, Weizhong Zhang, Wen Jiang, Anil Kumar, Shiyi Zhou, Zhengwei Cao, Shuyue Zhan, Wei Yang, Rui Liu, Yong Teng, Jin Xie

**Affiliations:** 1 Department of Chemistry, University of Georgia, Athens, GA 30602 USA; 2 Department of Hematology and Medical Oncology, Winship Cancer Institute, Emory University School of Medicine, Atlanta, GA 30322 USA

**Keywords:** Photodynamic therapy, Immunotherapy, Cancer, IDO, Ferritin, Nanoparticles

## Abstract

**Background:**

Photodynamic therapy (PDT) may elicit antitumor immune response in addition to killing cancer cells. However, PDT as a monotherapy often fails to induce a strong immunity. Immune checkpoint inhibitors, which selectively block regulatory axes, may be used in combination with PDT to improve treatment outcomes. Indoleamine 2,3-dioxygenase (IDO) is an immunoregulatory enzyme and an important meditator of tumor immune escape. Combination therapy with PDT and IDO-targeted immune checkpoint blockage is promising but has been seldom been explored.

**Methods:**

Herein we report a composite nanoparticle that allows for simultaneous delivery of photosensitizer and IDO inhibitor. Briefly, we separately load ZnF_16_Pc, a photosensitizer, and NLG919, an indoleamine 2,3-dioxygenase (IDO) inhibitor, into ferritin and poly(lactide-*co*-glycolic)-*block*-poly(ethylene glycol) (PEG-PLGA) nanoparticles; we then conjugate these two compartments to form a composite nanoparticle referred to as PPF NPs. We tested combination treatment with PPF NPs first in vitro and then in vivo in B16F10-tumor bearing C57/BL6 mice.

**Results:**

Our results showed that PPF NPs can efficiently encapsulate both ZnF_16_Pc and NLG919. In vivo studies found that the combination treatment led to significantly improved tumor suppression and animal survival. Moreover, the treatment increased tumor infiltration of CD8^+^ T cells, while reducing frequencies of MDSCs and Tregs. 30% of the animals showed complete tumor eradication, and they successfully rejected a second tumor inoculation. Overall, our studies introduce a unique composite nanoplatform that allows for co-delivery of photosensitizer and IDO inhibitor with minimal inter-species interference, which is ideal for combination therapy.

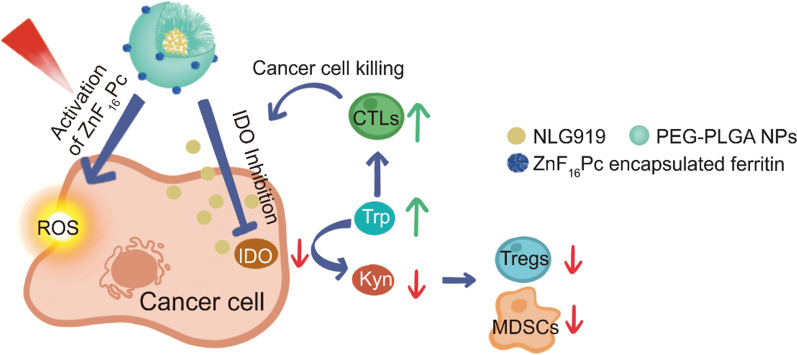

**Supplementary Information:**

The online version contains supplementary material available at 10.1186/s12951-021-00919-z.

## Background

Photodynamic therapy or PDT has been extensively tested for cancer treatment. During PDT, a large amount of reactive oxygen species such as singlet oxygen are produced, which cause physical damage to cancer cells or tumor vasculature [1]. PDT also induces immunogenic cell death (ICD), eliciting an antitumor immunity that benefits long-term tumor management [2]. However, PDT as a monotherapy is associated with a relatively high recurrence rate. While the reasons are multi-fold, one factor is that PDT may cause excessive inflammation that induces immunosuppressive mechanisms [2–4], leading to cancer cell immune escape and repopulation. Immune checkpoint inhibitors, which can selectively block related regulatory axes, may be used in combination with PDT to improve therapy outcomes [5–7]. For instance, several groups, including us, have demonstrated that PDT in conjugation with anti-PD-1/PD-L1/CTLA4 antibodies augments immune response [8, 9], facilitating suppression of both primary and distant tumors [10].

Indoleamine 2,3-dioxygenase (IDO) is another immune checkpoint frequently implicated in tumor immunosuppression [2]. IDO is a monomeric, heme-containing enzyme that metabolizes tryptophan (Trp) to kynurenine (Kyn). IDO is frequently upregulated in both cancer cells and host antigen-presenting cells (APCs) [11]. IDO overexpression leads to the depletion of Trp that is essential to the survival and functions of effector T cells, causing their G1 cycle arrest and apoptosis [11, 12]. Meanwhile, Kyn, the metabolite, can promote the differentiation and activation of regulatory T cells (Tregs) [13], facilitating the recruitment of myeloid-derived suppressor cells (MDSCs), culminating in a tolerogenic tumor microenvironment (TME) [11, 13, 14]. Multiple molecule IDO inhibitors have been developed and tested in pre-clinical and clinical studies [15]. PDT may induce the secretion of interferon γ (IFN-γ) and tumor necrosis factor α (TNF-α), leading to increased infiltration of Tregs and expansion of MDSCs with elevated IDO expression. Combining with IDO inhibition may promote PDT-induced antitumor immunity. The concept, however, has only recently been tested [2].

**Fig. 1:**
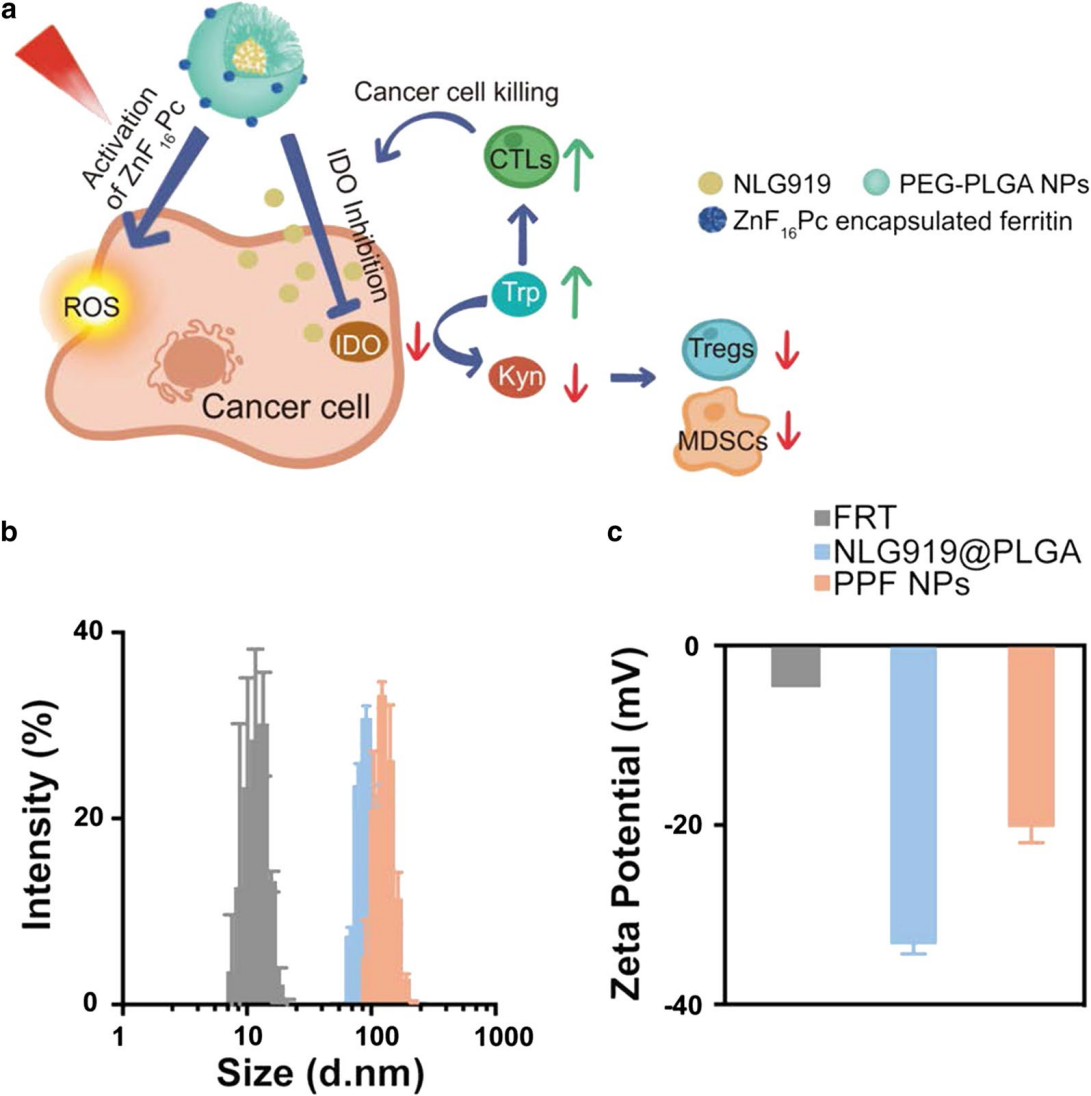
Preparation of characterizations of PPF NPs. **a** Schematic illustration showing the mechanism behind PPF NPs enabled immunotherapy. The PPF conjugate contains two nanocompartments, which are FRT and PLGA NPs. These two compartments are separately encapsulated with ZnF_16_Pc and NLG919, and covalently linked. Under photo-irradiation, ZnF_16_Pc is activated, leading to cancer cell death and release of tumor associated antigens. Meanwhile, NLG919 causes the suppression of IDO and restored the Trp/Kyn balance; this results in reduced tumor infiltration of Tregs and MDSCs and increased cytotoxic T cell activity, leading to enhanced antitumor immunity. **b** Hydrodynamic sizes of FRT, NLG919@PLGA and PPF NPs, measured by DLS. **c** Zeta potential of FRT, NLG919@PLGA and PPF NPs

Herein, we report a composite, core/satellite nanoparticle that allows for co-delivery of photosensitizer and IDO inhibitor for combination PDT and immune checkpoint blockade (ICB) therapy. Photosensitizers and IDO inhibitors belong to two categories of therapeutics that have different physiochemical and pharmacokinetic properties. While it is possible to load both types of therapeutics onto one nanoplatform, the drugs may interfere with each other and negatively affect loading and release. To solve the issue, we construct a composite nanostructure where zinc hexadecafluoro-phthalocyanine (ZnF_16_Pc) and NLG919 are encapsulated into separate nanocompartments. More specifically, ZnF_16_Pc, a near-infrared photosensitizer (ex: ~ 670 nm) is encapsulated into ferritin (FRT) protein cage, whereas NLG919, a potent enzymatic inhibitor of IDO, is encapsulated into poly(lactide-*co*-glycolic)-*block*-poly(ethylene glycol) (PEG-PLGA) nanoparticles. The two particles are covalently conjugated to form a composite nanoparticle (Fig. 1a) [15]. While ZnF_16_Pc is minimally released from ferritin thus enabling steadfast production of reactive oxygen species (ROS) under photo-irradiation [16, 17], NLG919 is released from PEG-PLGA nanoparticles in a controlled fashion to allow for sustained inhibition of IDO post PDT. We administered the resulting, PEG-PLGA and FRT composite nanoparticles (hereafter referred to as PPF NPs), into B16F10-tumor-bearing mice to examine their tumor suppression efficacy and antitumor immunity.

## Results and discussion

Heavy-chain ferritin was prepared and purified according to our published protocol [18]. ZnF_16_Pc was encapsulated into ferritin through a pH-mediated disassembly-and-reassembly approach, and purified on a desalting column to remove unbound ZnF_16_Pc [17, 19]. The resulting, ZnF_16_Pc encapsulated ferritin or ZnF_16_Pc@FRT, contains 40 wt% ZnF_16_Pc and has a diameter of ~ 12 nm. Meanwhile, NLG919 was encapsulated into polymeric nanoparticles made of PLGA-*b*-PEG-COOH (Mn: 7000 Da for PLGA and 1000 Da for PEG) through nanoprecipitation (Additional file 1: Figures S1, S2). This yielded NLG919-encapsulated PEG-PLGA nanoparticles (NLG919@PLGA NPs) with a loading rate of 6.63 wt%.

**Fig. 2:**
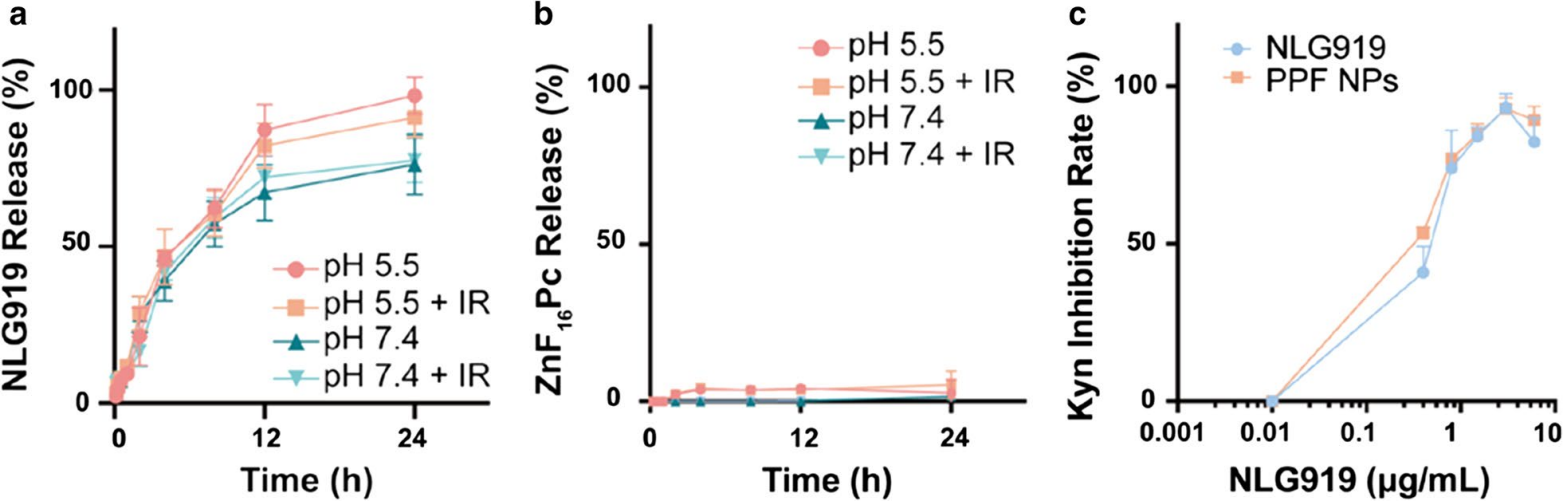
Drug release and IDO inhibition efficiency. **a**, **b** Drug release kinetics, tested with PPF NPs in buffer solutions with pH 5.5 and 7.4 in the presence and absence of photo-irradiation (IR). **a** NLG919 release profiles. **b** ZnF_16_Pc release profiles. **c** IDO inhibition, examined by measuring Kyn released from HeLa cells. Hela cells were first treated with IFN-γ along with either free NLG919 or PPF NPs. Kyn in the supernatant was quantified 48 h later, and compared to PBS-treated cells. The experiments were conducted in triplicate. Values are presented as means ± SD

To construct PPF core/satellite nanoparticles, the surface carboxyl groups of NLG919@PLGA NPs were activated by EDC/NHS chemistry; the resulting nanoparticles were mixed with ZnF_16_Pc@FRT to form a covalent linkage. The ratio between the two nanocompartments were adjusted so that in the final conjugate (i.e. PPF NPs), the NLG919 to ZnF_16_Pc ratio was ~ 7:1; this ratio was chosen based on the effective doses of the two therapeutics according to previous studies [17, 20]. Transmission electron microscopy (TEM) confirmed the coupling (Additional file 1: Figure S3). Dynamic light scattering (DLS) found an increased hydrodynamic diameter from 112.9 ± 0.7 to 144.0 ± 1.3 nm (Fig. 1b). This was accompanied by a surface charge increase from − 33.30 ± 1.03 to − 20.23 ± 1.72 mV (Fig. 1c), also confirming the conjugation.

We also studied drug release using UV–vis spectrophotometry. The release of NLG919 from PPF NPs was relatively slow at pH 7.4 (77.5% at 24 h, Fig. 2a), and accelerated under acidic conditions (98.2% at 24 h, Fig. 2a). Meanwhile, ZnF_16_Pc release was minimal at both neutral and acidic pH (Fig. 2b), which is consistent with our previous observations [19]. Notably, photo-irradiation did not significantly accelerate the release of either drug molecules (Fig. 2a, b).

We then evaluated the cytotoxicity of PPF NPs in B16F10 cells, which are a murine melanoma cell line. Briefly, B16F10 cells were incubated with PPF NPs (25 µg/mL, ZnF_16_Pc concentration) in the dark and irradiated at 4 h (671 nm, 0.1 W/cm^2^, 200 s). PPF NPs in the absence of radiation showed low toxicity (Additional file 1: Figure S4). Under radiation, there was a significant viability drop, which is attributed to PDT. We also evaluated the IDO inhibition efficacy of PPF NPs. Because B16F16 cells have a relatively low IDO expression, this was examined in HeLa cells, which express a high level of IDO and are commonly used for in vitro IDO inhibition assessment [20–22]. Briefly, HeLa cells were treated with IFN-γ to induce IDO expression, followed by incubation with PPF NPs for 48 h; the amounts of Kyn in the culturing medium were quantified using a colorimetric assay (Fig. 2c) [20]. PPF NPs showed efficient and concentration-dependent inhibition of Kyn production. The EC_50_ is 1.25 µg/mL, which is comparable to free NLG919 (Fig. 2c).

We next evaluated the therapeutic effects of PPF NPs in vivo in B16F10 tumor bearing C57BL/6 mice. We intratumorally (i.t.) injected PPF NPs (2.5 mg/kg NLG919, equivalent to 0.375 mg/kg ZnF_16_Pc) into the animals (n = 10). This was followed by photo-irradiation with a 671-nm laser (0.3 W/cm^2^, 15 min) applied to tumors at 4 h (Day 0, the treatment group was denoted as PPF + IR). Two more treatments were applied on Day 3 and 6. For comparison, NLG919@PLGA NPs in the absence of irradiation (NLG919@PLGA, n = 10) and ZnF_16_Pc@FRT plus irradiation (ZnF_16_Pc@FRT + IR, n = 10) were also tested. PBS only in the absence of irradiation was tested as a control (PBS, n = 8).

**Fig. 3:**
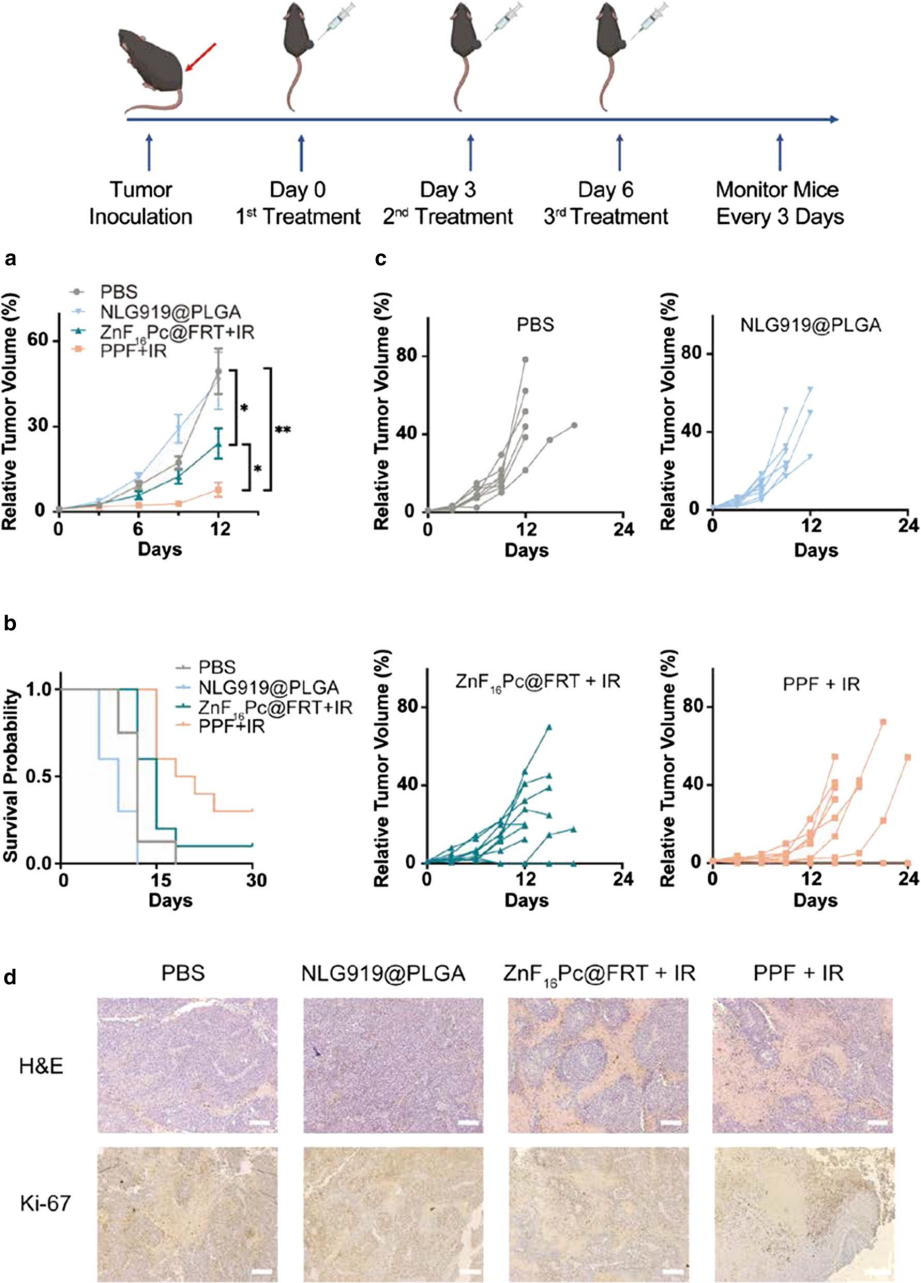
In vivo treatment efficacy, tested in C57BL/6 mice bearing B16F10 tumors. The animals were i.t. injected with PPF NPs and irradiated with a 671-nm laser at 4 h (PPF + IR). A total of three doses of treatment were given 2 days apart. PBS, NLG919@PLGA NPs in the absence of irradiation (NLG919@PLGA), and ZnF_16_Pc@FRT plus irradiation (ZnF_16_Pc@FRT + IR) were tested for comparison (n = 8–10). **a** Relative tumor volume change. Values are presented as means ± SEM. * p < 0.05, ** p < 0.01. **b** Animal survival. **c** Relative volume change for individual tumors from different treatment groups. **d** H&E and Ki-67 staining with tumor samples taken from all treatment groups. Scale bars, 100 μm

ZnF_16_Pc@FRT + IR caused a modest tumor suppression, inhibiting tumor growth by 51.27% relative to the PBS control group on Day 12 (Fig. 3a). However, this was followed by a rapid tumor rebound (Fig. 3a, c), with 40% of the animals reaching a humane endpoint point by Day 15. NLG919@PLGA failed to suppress tumor growth but rather promoted it in the short-term, although the difference was insignificant between the NLG919@PLGA and PBS groups (*p* = 0.81, Fig. 3a, c). All animals in the NLG919@PLGA group either died or had to be euthanized by Day 12. As a comparison, PPF + IR resulted in remarkable tumor suppression (84.19% on Day 12, Fig. 3a, c). 30% of the mice in the PPF + IR group showed complete tumor eradication and remained alive after 6 months. For dead or euthanized animals, we harvested their tumors and major organ tissues for histology analysis. H&E and Ki-67 staining confirmed efficient cancer cell killing and tumor growth suppression in the PPF + IR group (Fig. 3d).

For animals showing complete tumor elimination in the PPF + IR group, a re-challenge study was performed to assess whether the treatment promoted anticancer immunity. Briefly, we injected live B16F10 cells into the opposite flank of these animals on Day 69 and monitored the tumor growth. All of the mice successfully rejected the second inoculation, and remained healthy through the remainder of the experiment (Additional file 1: Figures S5, S6). As a comparison, the only surviving animal from the ZnF_16_Pc@FRT + IR group succumbed to the second inoculation on Day 84. We euthanized animals from the PPF + IR group on Day 159, and analyzed memory T cells by flow cytometry. We found that both central (CD3^+^CD8^+^CD62L^+^CD44^+^) and effector memory T cell (CD3^+^CD8^+^CD62L^−^CD44^+^) populations were significantly increased relative to naïve mice (Additional file 1: Figure S7a, b). These results suggested that animals treated with PPF + IR developed a strong antitumor immunity.

**Fig. 4:**
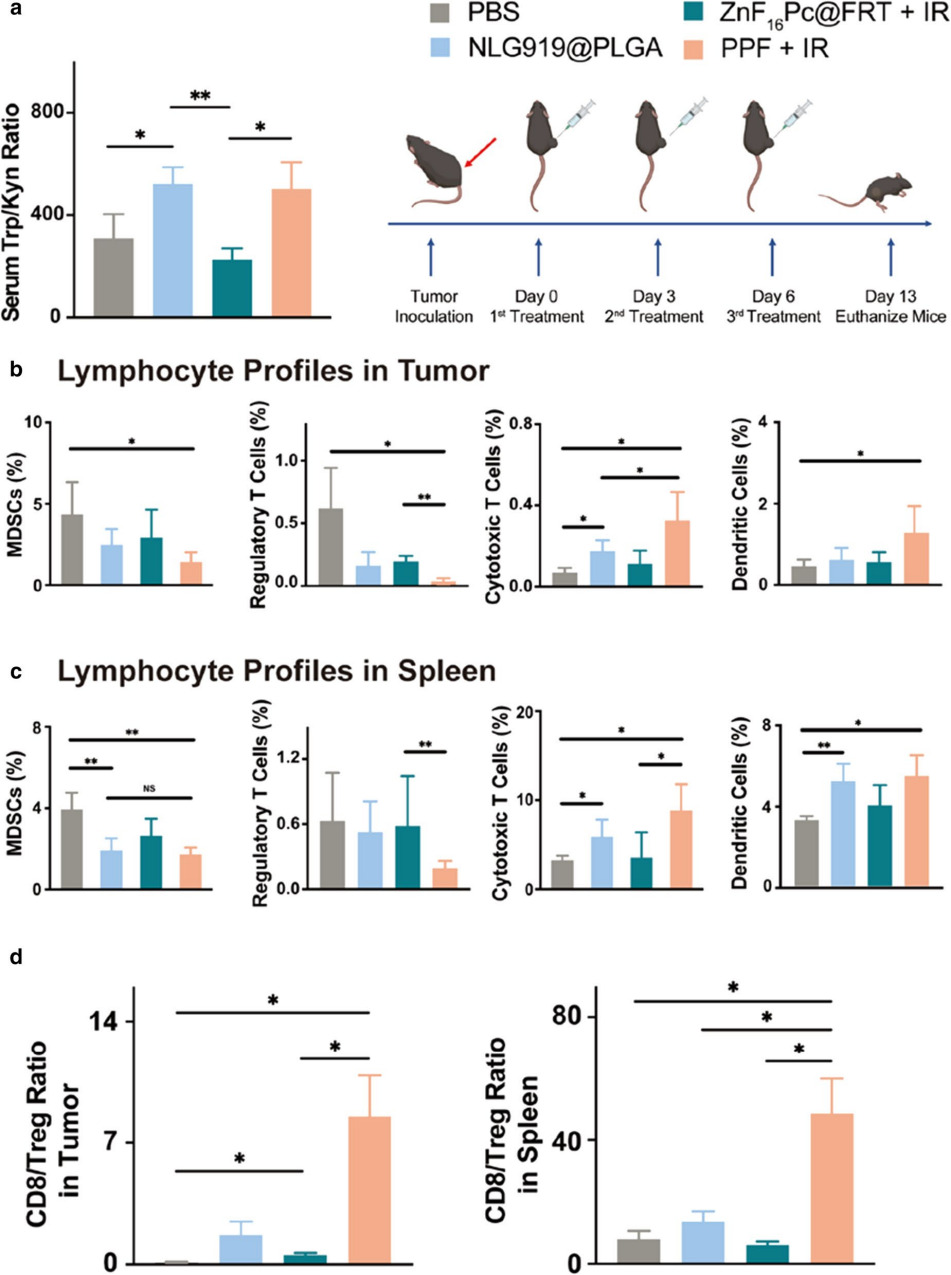
Antitumor immune response elicited by the combination therapy. **a** Serum Trp/Kyn ratios, measured by LC/MS. Blood samples were taken from animals 1 day after the treatments. **b**, **c** Lymphocyte frequencies in tumor (**c**) and spleen (**d**). Tissues were harvested 7 days after the third treatment. **d** CD8/Treg ratios in tumor (left) and spleen (right) tissues. Values are presented as means ± SD. *p < 0.05, **p < 0.01

To better understand treatment-induced immune responses, in a separate study, we treated animals with the same regimens (PPF + IR, PP, ZnF_16_Pc@FRT + IR, and PBS, single dose) and euthanized them 1 day after the treatment. We collected blood samples and analyzed serum concentrations of Trp and Kyn by LC–MS. We found that Trp/Kyn ratio was slightly reduced in the ZnF_16_Pc@FRT + IR group relative to the PBS control (Fig. 4a, p = 0.28), which is attributable to PDT-induced IDO upregulation. In both NLG919@PLGA and PPF + IR groups, Trp/Kyn ratios were significantly increased (Fig. 4a), which is owing to NLG919-based IDO inhibition.

We then repeated the therapy study but euthanized animals 7 days after the third treatment and collected tumor and spleen tissues to examine lymphocyte profiles. Compared to the PBS and ZnF_16_Pc@FRT groups, there was a significant decrease of MDSCs (Gr-1^+^CD11b^+^) population in tumors treated with NLG919@PLGA (Fig. 4b). PPF + IR led to an even greater reduction in the MDSCs population. More remarkable reduction was observed with Tregs (CD3^+^CD4^+^FoxP3^+^), whose abundance was decreased by 74.03 and 68.15%, respectively, in the NLG919@PLGA and ZnF_16_Pc@FRT + IR groups, and by 95. 97% in the PPF + IR group (Fig. 4b). Similar trends were observed in spleen tissue samples (Fig. 4c). Meanwhile, PPF + IR led to a significant increase in the dendritic cell (CD11c^+^) and cytotoxic T cell (CD3^+^CD8^+^) populations in both tumor and spleen samples (Fig. 4b, c). CD8/Treg ratio was dramatically increased from 54.52% in the ZnF_16_Pc@FRT + IR group to 848.43% in the PPF + IR group in tumor (Fig. 4d). Overall, these studies confirm that combination PDT and ICB with PPF NPs plus photo-irradiation induced a strong anticancer immunity.

**Fig. 5:**
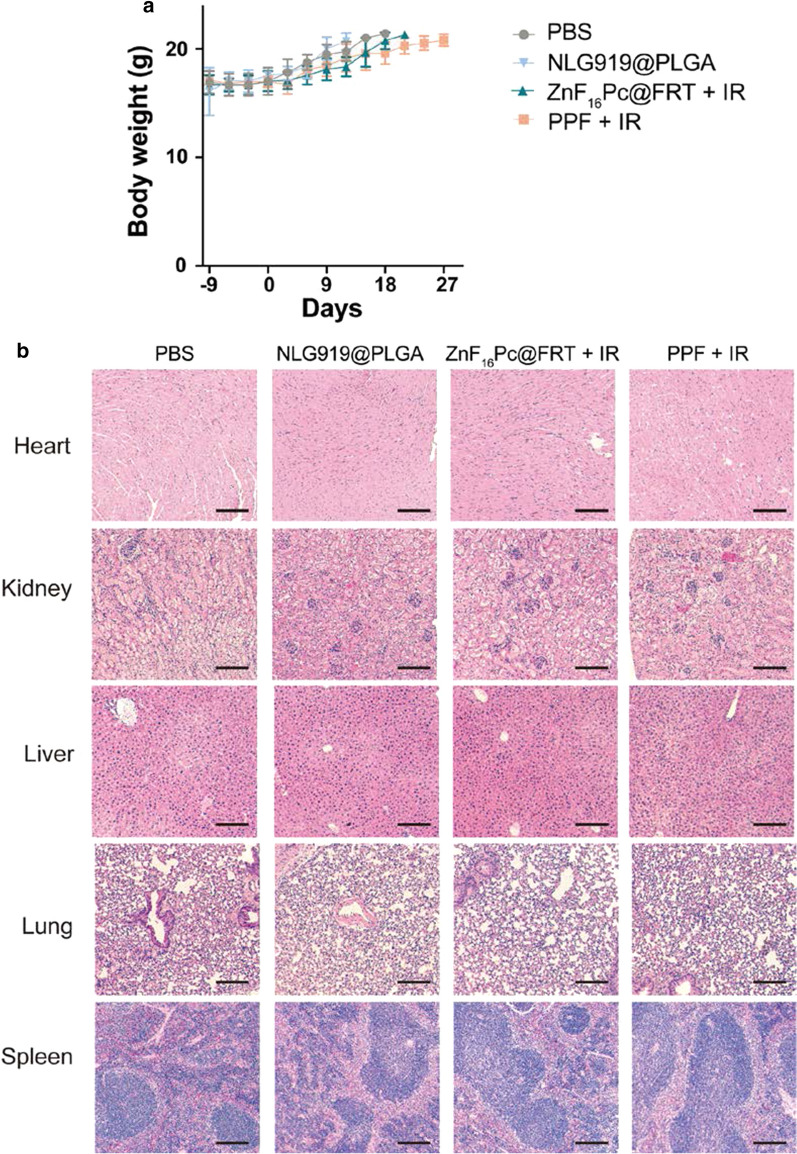
Potential side effects caused by the combination treatment. **a** Body weight curves. No significant body weight loss was observed throughout the study. **b** H&E staining of normal tissues, including the heart, kidney, liver, lung, and spleen. No signs of toxicity were detected. Scale bars, 100 μm

Meanwhile, the treatment was well tolerated by the animals. There were no signs of actuate toxicity after PDT. No body weight loss was observed throughout the studies (Fig. 5a). We also harvested tissues from normal organs, such as the heart, kidney, liver, lung, and spleen, and H&E staining found no signs of toxicity in the PPF + IR group (Fig. 5b).

## Conclusions

PDT can cause ICD, promoting recruitment and activation of innate immune cells such as dendritic cells [10]. This in turn may enhance antigen cross-presentation to T cells, stimulating specific antitumor immune responses. However, tumors may hijack immunoregulatory mechanisms to subvert PDT-induced immunity. Taking the current study for instance, ZnF_16_Pc@FRT + IR only caused a modest benefit in tumor suppression and animal survival, and failed to induce an abscopal effect. As a comparison, PPF NPs enable combination PDT and ICB therapy that significantly improves tumor management, resulting in complete tumor eradication in 30% of the treated animals and protecting them from a subsequent live cell challenge. Our unique composite nanoparticle is considered an important factor for effective treatment, for it enables efficient PDT and controlled release of IDO inhibitors. In current studies, nanoparticles were topically applied, which is suitable for melanoma therapy. It is postulated that this strategy can be used to treat stage III and recurrent melanoma, eliciting an antitumor immunity that not only eradicates local or residual cancer cells after surgery but also prevents metastasis. Notably, melanoma is relatively resistant to PDT due to the absorbance and anti-oxidant effects of melanin [23]. It is possible that switching to a photosensitizer with even longer excitation wavelength may improve the treatment. PPF nanoparticles may be systemically administered for treatment of other cancer types such as prostate and head and neck cancer. For these applications, the pharmacokinetics of the nanoparticles need to be re-assessed. PPF treatment may also synergize with anti-PD-1/PD-L1 antibodies that block other immunoregulatory pathways. These possibilities will be explored in future studies.

## Methods

### Materials

PLGA-COOH (MW: 7000, Lactel; cat#: B6013-1G); *N*-Hydroxysuccinimide (NHS) (Sigma Aldrich; cat#: 56480-25G); *N*-Ethyl-3-(3-dimethylaminopropyl)carbodiimide (EDC) (Sigma Aldrich; cat#: E7750); Dichloromethane (DCM) (Sigma Aldrich; cat#: 270997); Methanol (Fisher Scientific; cat#: A456-4); Diethyl Ether (Sigma Aldrich; cat#: 91238); NH2-PEG-COOH (MW: 1000, Biochempeg Scientific; cat#: HE005017-1 K); *N*,*N*-Diisopropylethylamine (DIEA) (Alfa Aesar; cat#: A11801); Acetonitrile (Sigma Aldrich; cat#: 271004-1 L); NLG919 (Advanced Chemblocks; cat#: L14096); Hydrochloric Acid (HCl) (J.T.Baker; cat#: 9535-03); Sodium Hydroxide (NaOH) (Fisher Scientific; cat#: S318-3); zinc hexadecafluoro-phthalocyanine (ZnF_16_Pc) (Sigma Aldrich; cat#: 444529); Dimethyl Sulfoxide (DMSO) (Sigma Aldrich; cat#: 276855); Uranyl Acetate 2% solution (Fisher Scientific, cat#: NC1085517); Illustra NAP-10 columns (GE Healthcare; cat#: 17-0854-02); Dulbecco’s Modified Eagle’s Medium (DMEM) (ATCC; cat#: 30-2002); Eagle’s Minimum Essential Medium (EMEM) (ATCC; cat#: 30-2003); Fetal Bovine Serum (ATCC; cat#: 30-2020); Penicillin–streptomycin (Sigma Aldrich; cat#: P0781-100 mL); 3-(4,5-dimethylthiazol-2-yl)-2,5-diphenyl Tetrazolium Bromide (MTT) (Sigma Aldrich; cat#: M2128); Human IFN-γ (R&D Systems; 285-IF); Trichloroacetic Acid (Fisher Scientific; cat#: ICN15259291); *p*-dimethylamino-benzaldehyde (Sigma Aldrich; cat#: 109,762); Glacial Acetic Acid (J.T.Baker; cat#: 9508-00).

### PLGA-***b***-PEG-COOH synthesis

PLGA-PEG-COOH polymer was synthesized using EDC/NHS chemistry. Briefly, 500 mg PLGA-COOH (0.071 mmol) and 109 mg EDC (0.7 mmol) were dissolved in 7 mL DCM, under magnetic stirring at room temperature for 10 min. Then, 160 mg NHS (1.4 mmol) was dissolved in 7 mL DCM, followed by dropwise addition into the above solution. The reaction was conducted overnight at room temperature. PLGA-NHS was washed with cold 1:1 methanol/diethyl ether mixture for three times, collected by centrifugation, and lyophilized. Then, 200 mg PLGA-NHS (0.028 mmol) was dissolved in 5 mL DCM under magnetic stirring at room temperature for 10 min. 30 mg NH_2_-PEG-COOH (0.03 mmol) and 60 µL DIEA (0.28 mmol) were dissolved in 2 mL DCM, and dropwise added into the PLGA-NHS solution. The reaction was conducted overnight. The conjugated PLGA-*b*-PEG-COOH polymer was washed with cold, 1:1 v/v methanol/diethyl ether mixture for three times, collected by centrifugation, and lyophilized. The product was stored at – 20 °C. Chemical structure was confirmed by 1 H-NMR on a Varian Mercury Plus 400 system.

### NLG919@PLGA nanoparticle synthesis

NLG919@PLGA NPs were synthesized through a nanoprecipitation method. Briefly, PLGA-*b*-PEG-COOH and NLG919 were first dissolved in acetonitrile; the final concentrations were 5 mg/mL for PLGA-*b*-PEG-COOH and 1.5 mg/mL for NLG919. The mixture was dropwise added into sterilized nanopure water with constant stirring for 2 h in fume hood. The resulting NLG919@PLGA NPs were collected on an amicon ultracentrifugation unit (MWCO 100 kDa) and washed 3–4 times with sterilized nanopure water. Finally, the purified NLG919@PLGA NPs were resuspended in sterilized nanopure water and kept at 4 °C.

### Preparation of ZnF_16_Pc@FRT

Protocols for ferritin (FRT) expression and purification were published before [17]. ZnF_16_Pc loading was achieved by following a published protocol with modification. Briefly, FRT was dispersed in PBS (pH 7.4), and the solution pH was reduced to 2.0 by adding 0.2 M HCl. ZnF_16_Pc (5 mg/mL in DMSO) was added into the above solution to reach a final FRT/ZnF_16_Pc w/w ratio of 5:1. After gently shaking at room temperature for 30 min, the pH of the mixture was slowly adjusted back to 7.4 by adding 1 M NaOH. NAP-10 column was used to remove unloaded ZnF_16_Pc.

### Preparation of PPF NPs

Briefly, NLG919@PLGA NPs (10 mg/mL) were resuspended in 3 mL sterilized nanopure water with constant stirring. Then, 720 µL EDC (10 mg/mL) was dropwise added, followed by the addition of 440 µL NHS (10 mg/mL), with constant stirring for 2 h under room temperature. The conjugates were collected by centrifugation for 3 times at 15 °C. The purified nanoparticles were suspended in 5 mL PBS (pH 7.4) with constant stirring at 4 °C in the dark, followed by the addition of ZnF_16_Pc@FRT (0.25 mg, based on FRT weight) into the solution. The mixture was stirred for 4 h. Finally, the conjugated nanoparticles were collected and purified by PBS (pH 7.4) for three times. PPF NPs were stored at 4 °C protected from light.

The ZnF_16_Pc to NLG919 ratio was adjusted to be ~ 1:7, based on previous research and experience. ZnF_16_Pc@FRT at a dose of 1.5 mg/kg of ZnF_16_Pc is commonly tested in tumor bearing mice with i.v. administration. According to our experience, about 10% of ZnF_16_Pc@FRT could accumulate in the tumor site. NLG919 at a dose of 25 mg/kg on mice with i.v. injection is normally used in cancer immunotherapy [20]. Polymeric nanoparticles are reported to accumulate in tumors at a rate of 3–5%. Based on our computation, a ZnF_16_Pc-to-NLG919 ratio of 1:7 is reasonable for combination therapy.

### Drug release

For drug release studies, 100 µL nanoparticle solution was loaded onto a dialysis unit, with or without irradiation (671 nm, 0.1 W/cm^2^ for 200 s). The unit was floated on top of a 1.1-mL buffer solution (pH 5.5 or 7.4). The system was positioned on an Eppendorf shaker set at 37 °C. At each time point (0.5, 1, 2, 4, 8, 12, 24, 36 and 48 h), 100 µL external solution was transferred into a 96-well plate. The drug contents were assessed by measuring the relevant absorbance (NLG919: 263 nm, ZnF_16_Pc: 700 nm) and comparing to pre-established standard curves.

### Cell culturing

B16F10 and HeLa cells were obtained from American Type Culture Collection (ATCC) and cultured in DMEM or EMEM medium supplemented with 10% fetal bovine serum and 1% penicillin–streptomycin. The cells were incubated humidly at 37 °C with 5% CO_2_.

### Cytotoxicity assays

B16F10 cells were seeded onto a 96-well plate at 8000 cells per well density and allowed to grow overnight. Nanoparticles of varied concentrations were added to the incubation medium. After 4 h, the cells were exposed to a 671-nm laser (0.1 W/cm^2^ for 200 s). Cells not exposed to photo-irradiation were tested for comparison. After another 12 h, cell viability was measured by MTT assay.

### IDO activity

Briefly, HeLa cells were seeded onto a 96-well plate at a density of 8000 cells per well and allowed to grow overnight. Recombinant human IFN-γ was added to each well to reach a final concentration of 50 ng/mL. Meantime, a gradient of NLG919@PLGA NPs or PPF NPs (NLG919 concentration 0, 0.4, 0.8, 1.5, 3, 6 µg/mL) were added to the incubation medium. After 48 h, 150 µL of the supernatants per well was transferred to a separate 96-well plate. 75 µL 30% trichloroacetic acid was added into each well and incubated with cells at 50 °C for 30 min, converting *N*-formylkynurenine to kynurenine. The supernatant was transferred to a new 96-well plate, mixed with equal volume of Ehrlich reagent (2% *p*-dimethylamino-benzaldehyde w/v in glacial acetic acid), and incubated for 10 min at room temperature. Reaction product was quantified by measuring 490-nm absorbance using a plate reader.

### Animal model

C57BL/6 mice were purchased from Envigo laboratories. The animal model was established by subcutaneously injecting 2 × 105 B16F10 cells into the right hind limb of each mouse. All of the experimental procedures were conducted following a protocol approved by the University of Georgia Institutional Animal Care and Use Committee.

### Therapy studies

C57BL/6 mice bearing B16F10 tumors were randomly divided into four groups (n = 8–10): (1) PBS, no irradiation; (2) NLG919@PLGA NPs (NLG919 2.5 mg/kg), no irradiation; (3) ZnF_16_Pc@FRT (ZnF_16_Pc 0.375 mg/kg), with irradiation; (4) PPF NPs (NLG919 2.5 mg/kg, ZnF_16_Pc 0.375 mg/kg), with irradiation. All mice received i.t. injection every 3 days for 3 times. For the ZnF_16_Pc@FRT + IR and PPF + IR groups, tumors received photo-irradiation 4 h after the injection (671 nm, 0.3 W/cm^2^ for 15 min). Tumor size and body weight were monitored every 3 days. Tumor volume was computed using formula: tumor volume = length × (width)^2^/2. After therapy, major organs as well as tumors were collected and sectioned into 8-µm slices for H&E and Ki-67 staining. For re-challenge studies, each of the surviving animals was subcutaneously injected with 1 × 105 B16F10 cells to the left hind limb. Tumor size and body weight of were monitored every 3 days.

### Serum tryptophan and kynurenine analysis

Trp/Kyn ratios in serum samples were examined by LC/MS on a Bruker Daltonics Impact II system. C57BL/6 mice bearing B16F10 tumor at ~ 50 mm^3^ were treated with single-dose of PBS, NLG919@PLGA NPs, ZnF_16_Pc@FRT + IR, or PPF + IR (NLG919 2.5 mg/kg, ZnF_16_Pc 0.375 mg/kg). One day after the treatment, the plasma samples were collected and mixed with methanol (plasma:methanol, 1:2.5, v/v) and centrifuged at 13,500 rpm for 15 min. Supernatants were collected and purified with TopTip for LC–MS quantification of kynurenine and tryptophan.

### Lymphocyte profiling studies

Seven days after the third treatment, animals were euthanized; spleen and tumors were harvested, and immune cell populations in the tissues was examined by flow cytometry. Briefly, single-cell suspensions from tissue samples were filtered and red blood cells were lysed. For extracellular staining, cells were incubated with the indicated combinations of antibodies (e.g. anti-CD11c, -Gr-1, -CD3, -CD8α, -CD4, and -CD45). For intracellular staining, cells were fixed and permeabilized immediately after cell surface staining according to manufacturer’s protocol (eBioscience). Antibodies (e.g. anti-Foxp3) were added into the permeabilization buffer and incubated with cells. Animals surviving from re-challenge studies were euthanized after 30 days, and spleen tissues were harvested for analysis of memory T cell populations. Immune cell populations were examined on a Beckman Coulter CytoFLEX system. Data analyzed was performed using FlowJo software.

### Statistical analysis

Comparison of multiple assays was performed using a one-way ANOVA test. Comparisons of only two groups was performed using a paired t-test. Significance was set at p < 0.05. All experiments were performed with at least three replicates unless specified otherwise. All the data is represented as mean ± S.D.

## Supplementary Information

**Additional file 1.** Additional Information includes a schematic illustration of nanoparticle preparation, additional experimental data for PLGA-*b*-PEG characterizations, nanoparticle cytotoxicity, tumor growth in the re-challenge study, animal survival, central and effector memory T cell abundance, and absorbance spectra.

## Data Availability

All data generated or analyzed during this study are included in this published article and the Additional Information.

## References

[1] Dolmans DE, Fukumura D, Jain RK (2003). Photodynamic therapy for cancer. Nat Rev Cancer.

[2] Wachowska M, Stachura J, Tonecka K, Fidyt K, Braniewska A, Sas Z, Kotula I, Rygiel TP, Boon L, Golab J, Muchowicz A (2020). Inhibition of IDO leads to IL-6-dependent systemic inflammation in mice when combined with photodynamic therapy. Cancer Immunol Immun.

[3] Wan J, Wu W, Che Y, Kang NN, Zhang RQ (2015). Low dose photodynamic-therapy induce immune escape of tumor cells in a HIF-1 alpha, dependent manner through PI3K/Akt pathway. Int Immunopharmacol.

[4] Milla Sanabria L, Rodriguez ME, Cogno IS, Rumie Vittar NB, Pansa MF, Lamberti MJ, Rivarola VA (2013). Direct and indirect photodynamic therapy effects on the cellular and molecular components of the tumor microenvironment. Biochim Biophys Acta.

[5] Dovedi SJ, Adlard AL, Lipowska-Bhalla G, McKenna C, Jones S, Cheadle EJ, Stratford IJ, Poon E, Morrow M, Stewart R, Jones H, Wilkinson RW, Honeychurch J, Illidge TM (2014). Acquired resistance to fractionated radiotherapy can be overcome by concurrent PD-L1 blockade. Cancer Res.

[6] Schaue D, Xie MW, Ratikan JA, McBride WH (2012). Regulatory T cells in radiotherapeutic responses. Front Oncol.

[7] Lamberti MJ, Mentucci FM, Roselli E, Araya P, Rivarola VA, Rumie Vittar NB, Maccioni M (2019). Photodynamic modulation of type 1 interferon pathway on melanoma cells promotes dendritic cell activation. Front Immunol.

[8] Xu J, Xu L, Wang C, Yang R, Zhuang Q, Han X, Dong Z, Zhu W, Peng R, Liu Z (2017). Near-infrared-triggered photodynamic therapy with multitasking upconversion nanoparticles in combination with checkpoint blockade for immunotherapy of colorectal cancer. ACS Nano.

[9] Chen SX, Ma M, Xue FF, Shen SZ, Chen Q, Kuang YC, Liang KC, Wang XL, Chen HR (2020). Construction of microneedle-assisted co-delivery platform and its combining photodynamic/immunotherapy. J Control Release.

[10] Zhou S, Li D, Lee C, Xie J (2020). Nanoparticle phototherapy in the era of cancer immunotherapy. Trends Chem.

[11] Holmgaard RB, Zamarin D, Li Y, Gasmi B, Munn DH, Allison JP, Merghoub T, Wolchok JD (2015). Tumor-expressed IDO recruits and activates MDSCs in a Treg-dependent manner. Cell Rep.

[12] Prendergast GC, Smith C, Thomas S, Mandik-Nayak L, Laury-Kleintop L, Metz R, Muller AJ (2014). Indoleamine 2,3-dioxygenase pathways of pathogenic inflammation and immune escape in cancer. Cancer Immunol Immunother.

[13] Mellor AL, Munn DH (2004). IDO expression by dendritic cells: tolerance and tryptophan catabolism. Nat Rev Immunol.

[14] Spranger S, Spaapen RM, Zha Y, Williams J, Meng Y, Ha TT, Gajewski TF (2013). Up-regulation of PD-L1, IDO, and T-regs in the melanoma tumor microenvironment is driven by CD8(+) T cells. Sci Transl Med.

[15] Nayak A, Hao Z, Sadek R, Vahanian N, Ramsey WJ, Kennedy E, Mautino M, Link C, Bourbo P, Dobbins R, Adams K, Diamond A, Marshall L, Munn DH, Janik J, Khleif SN (2014). A phase I study of NLG919 for adult patients with recurrent advanced solid tumors. J Immunother Cancer.

[16] Zhen ZP, Tang W, Todd T, Xie J (2014). Ferritins as nanoplatforms for imaging and drug delivery. Expert Opin Drug Del.

[17] Zhen Z, Tang W, Guo C, Chen H, Lin X, Liu G, Fei B, Chen X, Xu B, Xie J (2013). Ferritin nanocages to encapsulate and deliver photosensitizers for efficient photodynamic therapy against cancer. ACS Nano.

[18] Lin X, Xie J, Niu G, Zhang F, Gao H, Yang M, Quan Q, Aronova MA, Zhang G, Lee S, Leapman R, Chen X (2011). Chimeric ferritin nanocages for multiple function loading and multimodal imaging. Nano Lett.

[19] Zhen Z, Tang W, Wang M, Zhou S, Wang H, Wu Z, Hao Z, Li Z, Liu L, Xie J (2017). Protein nanocage mediated fibroblast-activation protein targeted photoimmunotherapy to enhance cytotoxic T cell infiltration and tumor control. Nano Lett.

[20] Chen YC, Xia R, Huang YX, Zhao WC, Li J, Zhang XL, Wang PC, Venkataramanan R, Fan J, Xie W, Ma XC, Lu BF, Li S (2016). An immunostimulatory dual-functional nanocarrier that improves cancer immunochemotherapy. Nat Commun.

[21] Liu X, Shin N, Koblish HK, Yang G, Wang Q, Wang K, Leffet L, Hansbury MJ, Thomas B, Rupar M, Waeltz P, Bowman KJ, Polam P, Sparks RB, Yue EW, Li Y, Wynn R, Fridman JS, Burn TC, Combs AP, Newton RC, Scherle PA (2010). Selective inhibition of IDO1 effectively regulates mediators of antitumor immunity. Blood.

[22] Hou DY, Muller AJ, Sharma MD, DuHadaway J, Banerjee T, Johnson M, Mellor AL, Prendergast GC, Munn DH (2007). Inhibition of indoleamine 2,3-dioxygenase in dendritic cells by stereoisomers of 1-methyl-tryptophan correlates with antitumor responses. Cancer Res.

[23] Huang YY, Vecchio D, Avci P, Yin R, Garcia-Diaz M, Hamblin MR (2013). Melanoma resistance to photodynamic therapy: new insights. Biol Chem.

