# Supplementary material for: Nanoconjugates to enhance PDT-mediated cancer immunotherapy by targeting the indoleamine-2,3-dioxygenase pathway
Source: J Nanobiotechnology. 2021 Jun 14;19:182. doi: 10.1186/s12951-021-00919-z (PMC8201842; doi:10.1186/s12951-021-00919-z)
Supplement: Supplementary file 1 — Additional file 1. Additional Information includes a schematic illustration of nanoparticle preparation, additional experimental data for PLGA-b-PEG characterizations, nanoparticle cytotoxicity, tumor growth in the re-challenge study, animal survival, central and effector memory T cell abundance, and absorbance spectra. [file 12951_2021_919_MOESM1_ESM.docx]

Additional File 1

Supplementary Information for

**Nanoconjugates to enhance PDT-mediated cancer immunotherapy by targeting the indoleamine-2,3-dioxygenase pathway**

Xueyuan Yang^1^, Weizhong Zhang^1^, Wen Jiang^1^, Anil Kumar^1^, Shiyi Zhou^1^, Zhengwei Cao^1^, Shuyue Zhan^1^, Wei Yang^1^, Rui Liu,^1^ Yong Teng,^2^ Jin Xie^1,*^

^1^ Department of Chemistry, University of Georgia, Athens, GA 30602, USA

^2^ Department of Hematology and Medical Oncology, Winship Cancer Institute, Emory University School of Medicine, Atlanta, GA, 30322, USA


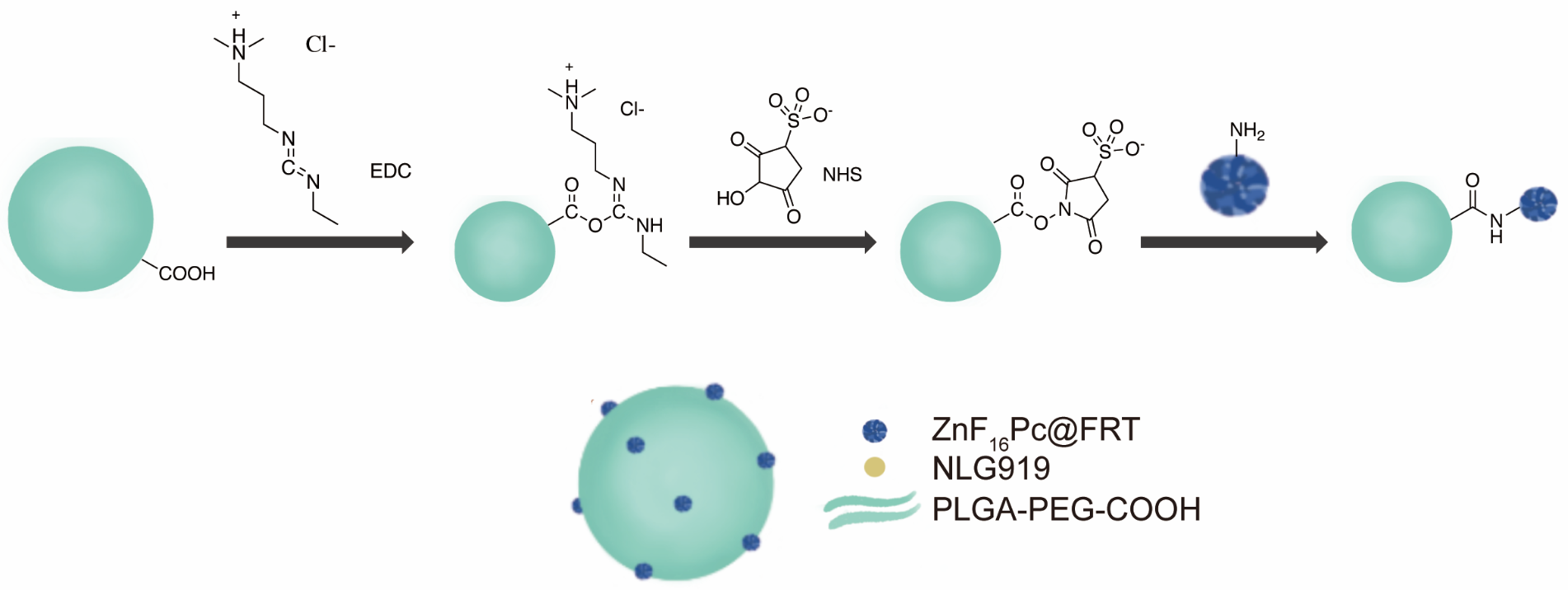


**Scheme S1:** Schematic illustration to show the preparation of PPF NPs.


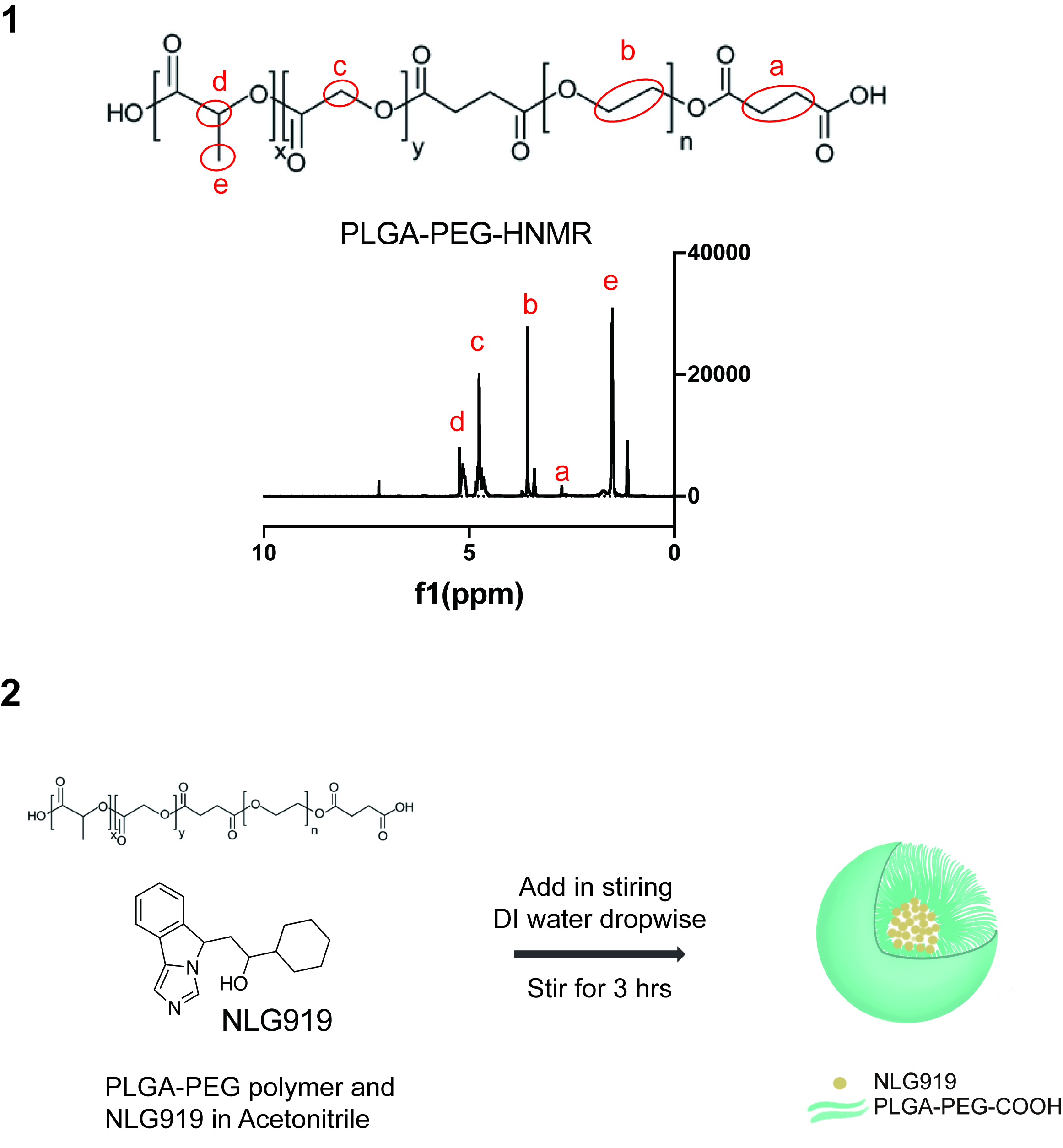


**Figure S1:** ^1^H-NMR spectrum of PLGA-b-PEG.


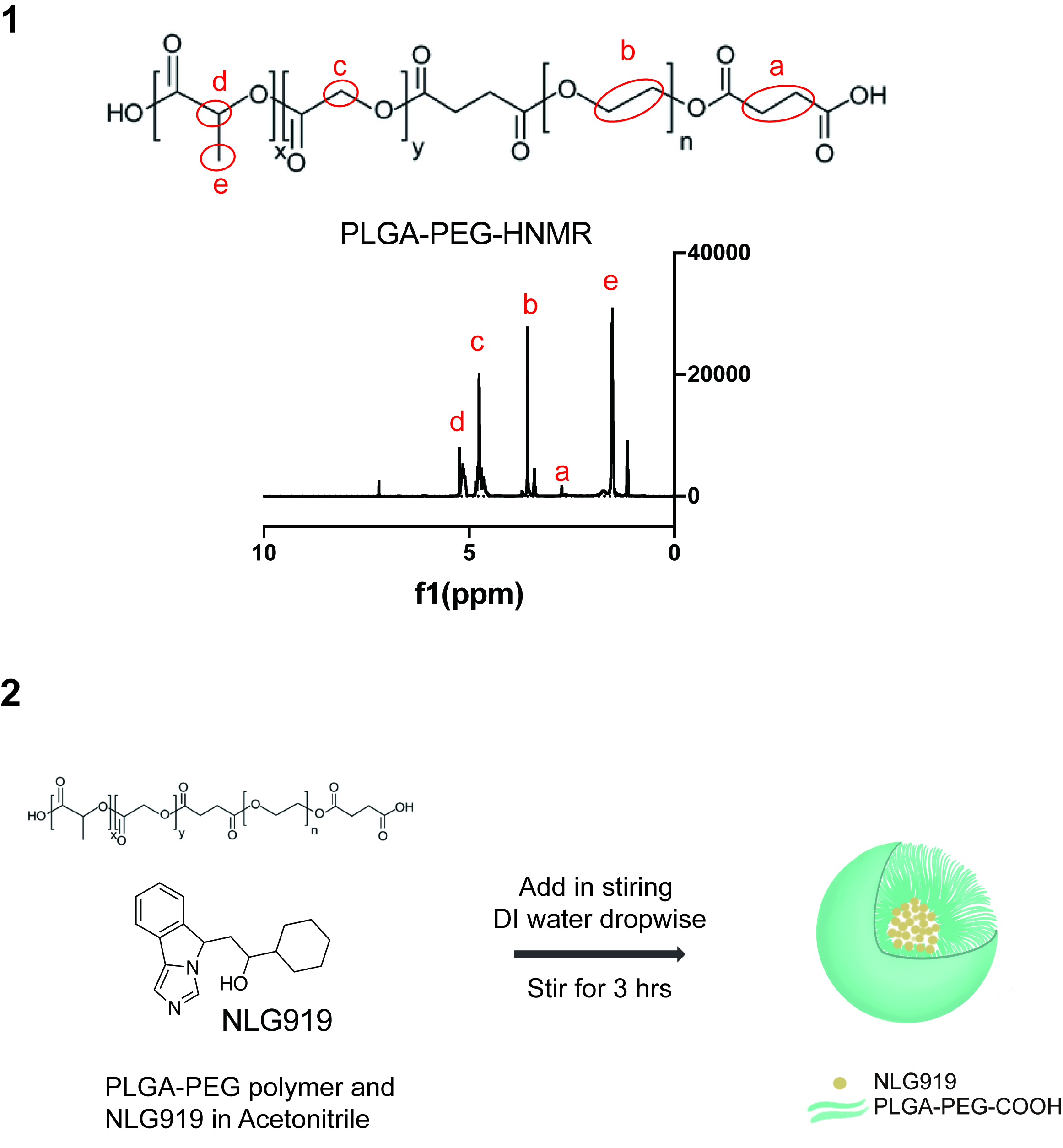


**Figure S2:** Schematic illustration to show the preparation of NLG919@PLGA NPs.


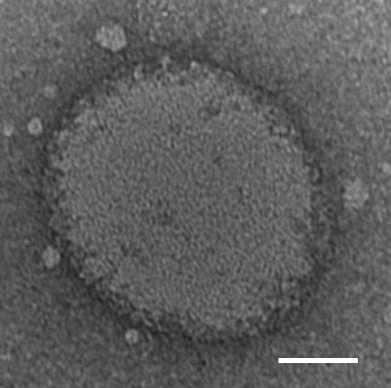


**Figure S3:** TEM image of PPF NPs. Scale bar, 50 nm.

**
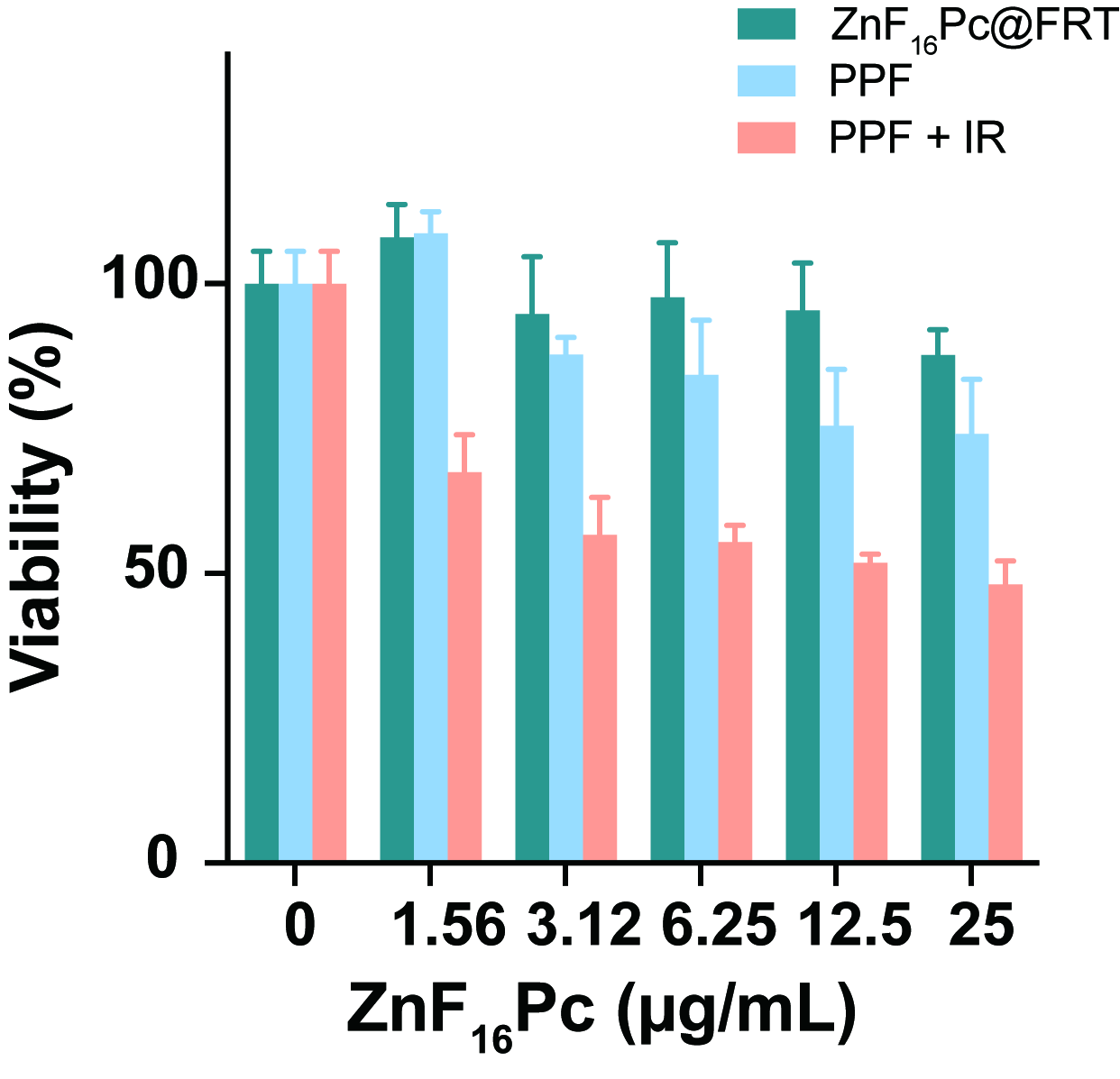
**

**Figure S4:** Cytotoxicity, measured with B16F10 cells by MTT assays. Cells were incubated with ZnF_16_Pc@FRT or PPF NPs for 4 h and then irradiated by a 671-nm laser (0.1 W/cm2 for 200 s). MTT assays were conducted 12 h later. *, p < 0.05; **, p < 0.01; ns, no significant difference.


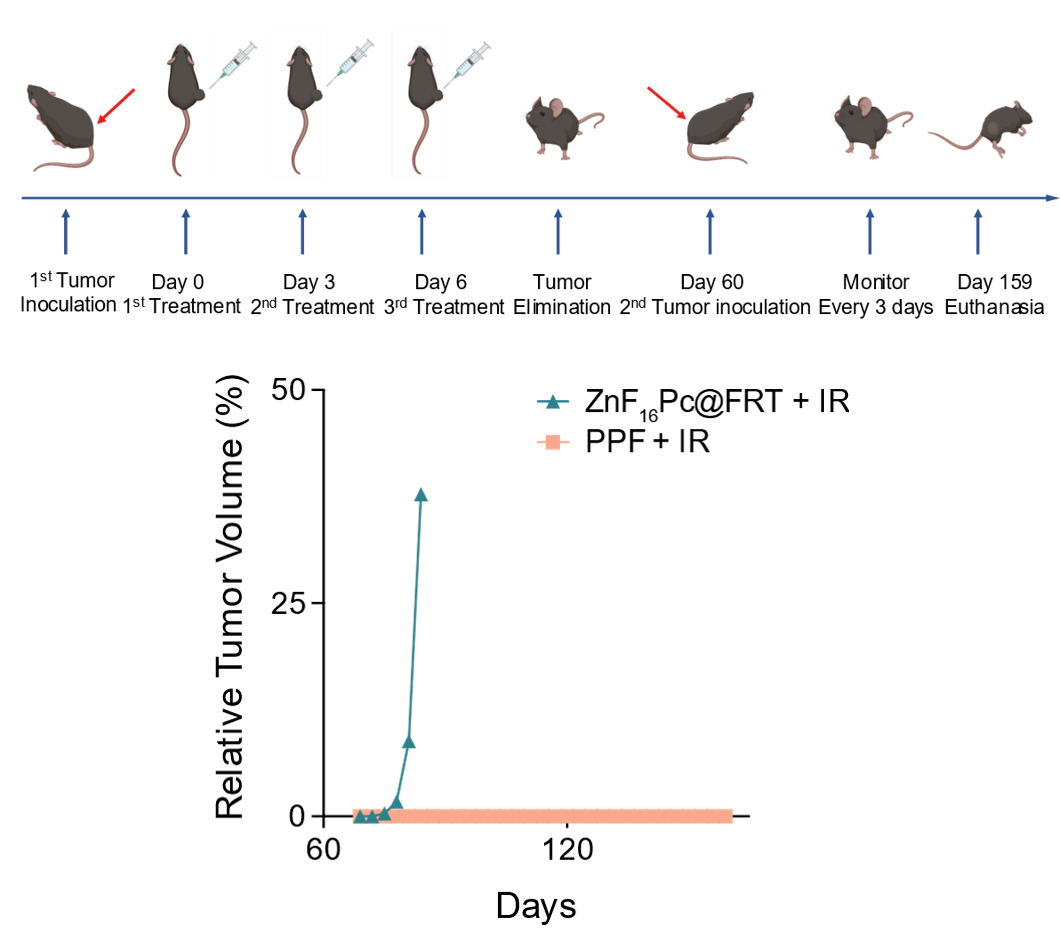


**Figure S5:** Relative tumor volume after live cell re-challenge. B16F10-tumor bearing mice surviving the initial ZnF_16_Pc@FRT+IR or PPF+IR treatments were inoculated with live B16F10 cancer cells to opposite flank of the mice on Day 69. The animals were monitored for another 90 days. All animals in the PPF+IR group remained alive and tumor-free throughout the study.


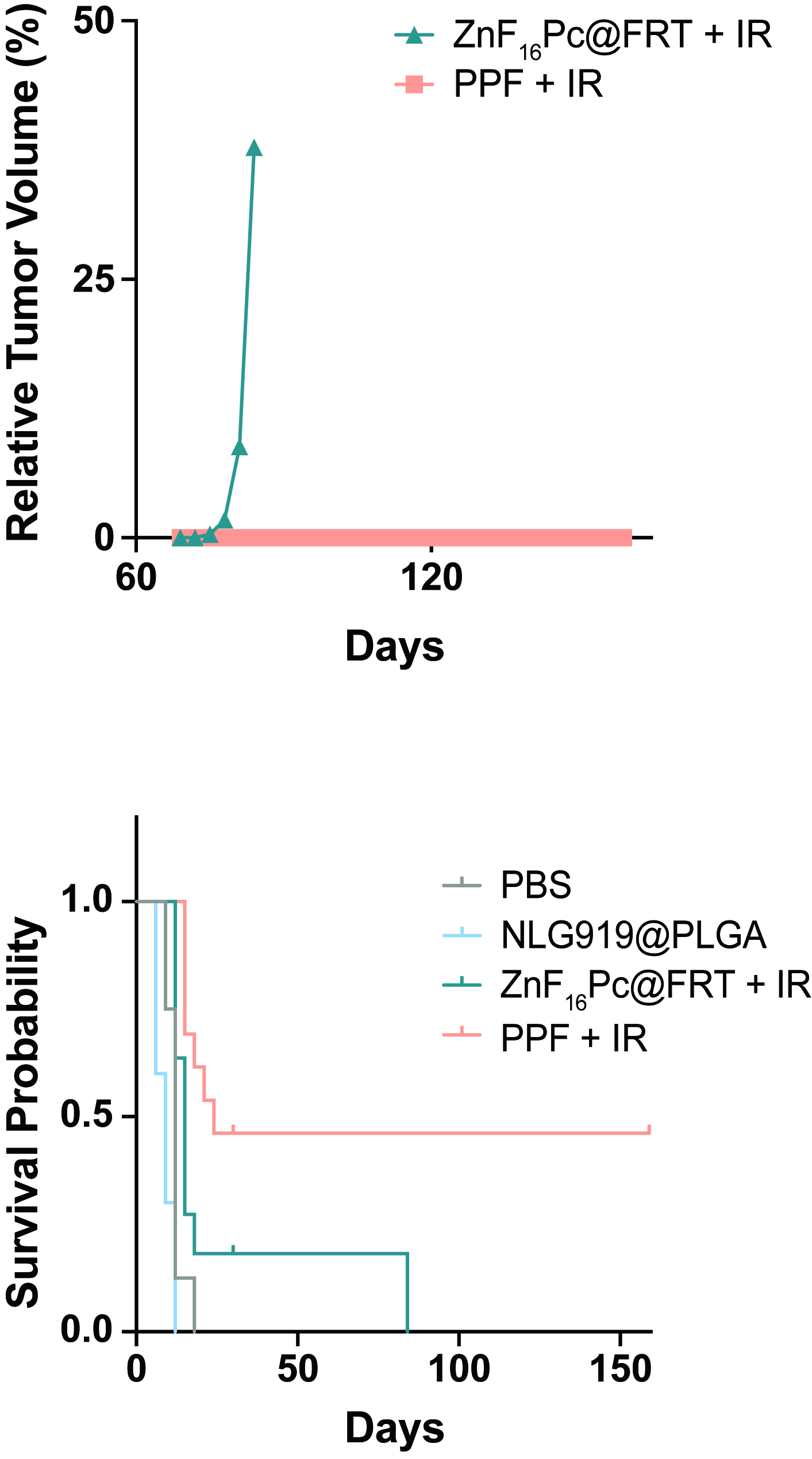


**Figure S6:** Animal survival throughout the experiment, including the re-challenge study.


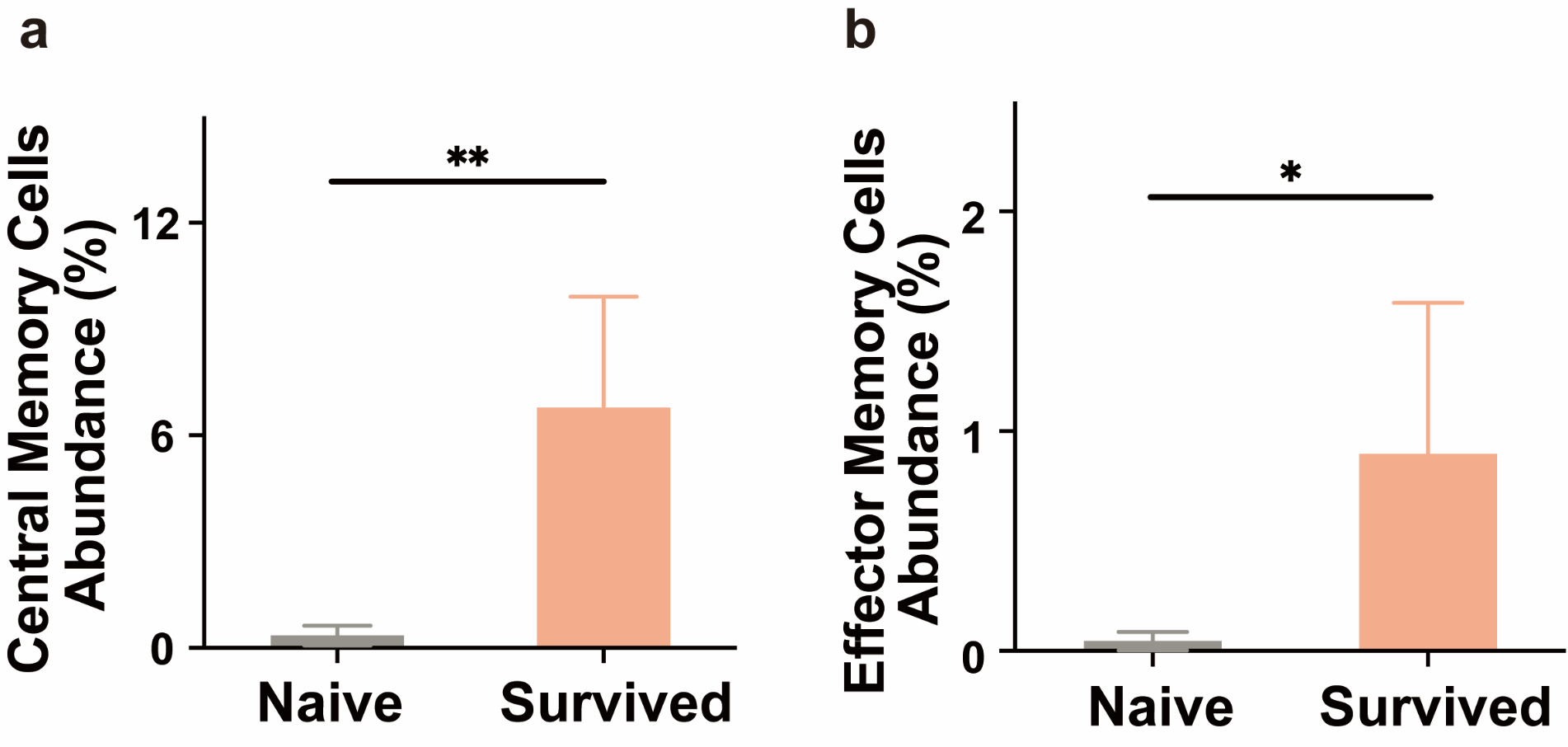


**Figure S7:** a,b) Central (a) and effector (b) memory T cells abundance, based on flow cytometry analysis of spleen tissues taken from animals treated with PPF+IR. **p* < 0.05, ***p* < 0.01; n=3. Spleen tissues from naïve animals were tested as a comparison.


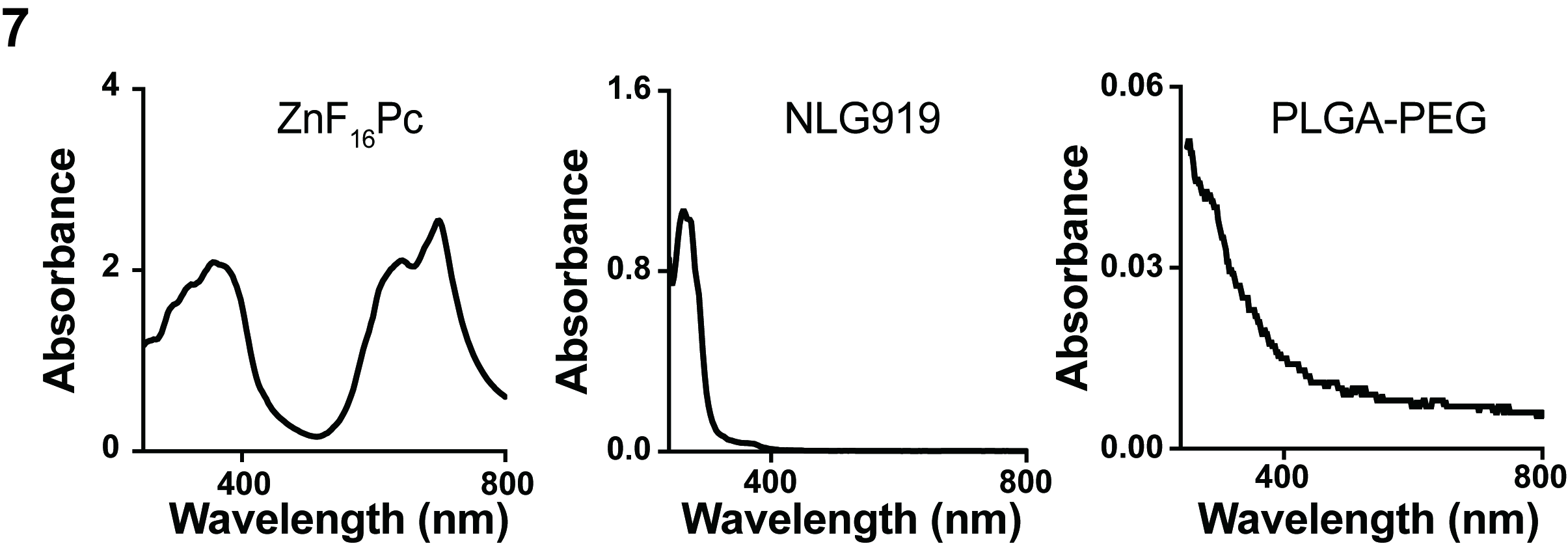


**Figure S8:** Absorbance spectra of ZnF_16_Pc, NLG919, and PLGA-*b*-PEG in DMSO.
